# Effects of Ambient Air Pollution Exposure on Olfaction: A Review

**DOI:** 10.1289/EHP136

**Published:** 2016-06-10

**Authors:** Gaurav S. Ajmani, Helen H. Suh, Jayant M. Pinto

**Affiliations:** 1 Pritzker School of Medicine, University of Chicago, Chicago, Illinois, USA; 2 Department of Health Sciences, Northeastern University, Boston, Massachusetts, USA; 3 Section of Otolaryngology-Head and Neck Surgery, Department of Surgery, University of Chicago, Chicago, Illinois, USA

## Abstract

**Background:**

Olfactory dysfunction affects millions of people worldwide. This sensory impairment is associated with neurodegenerative disease and significantly decreased quality of life. Exposure to airborne pollutants has been implicated in olfactory decline, likely due to the anatomic susceptibility of the olfactory nerve to the environment. Historically, studies have focused on occupational exposures, but more recent studies have considered effects from exposure to ambient air pollutants.

**Objectives:**

To examine all relevant human data evaluating a link between ambient pollution exposure and olfaction and to review supporting animal data in order to examine potential mechanisms for pollution-associated olfactory loss.

**Methods:**

We identified and reviewed relevant articles from 1950 to 2015 using PubMed and Web of Science and focusing on human epidemiologic and pathophysiologic studies. Animal studies were included only to support pertinent data on humans. We reviewed findings from these studies evaluating a relationship between environmental pollutant exposure and olfactory function.

**Results:**

We identified and reviewed 17 articles, with 1 additional article added from a bibliography search, for a total of 18 human studies. There is evidence in human epidemiologic and pathologic studies that increased exposure to ambient air pollutants is associated with olfactory dysfunction. However, most studies have used proxies for pollution exposure in small samples of convenience. Human pathologic studies, with supporting animal work, have also shown that air pollution can contact the olfactory epithelium, translocate to the olfactory bulb, and migrate to the olfactory cortex. Pollutants can deposit at each location, causing direct damage and disruption of tissue morphology or inducing local inflammation and cellular stress responses.

**Conclusions:**

Ambient air pollution may impact human olfactory function. Additional studies are needed to examine air pollution–related olfactory impacts on the general population using measured pollution exposures and to link pollution exposure with olfactory dysfunction and related pathology.

**Citation:**

Ajmani GS, Suh HH, Pinto JM. 2016. Effects of ambient air pollution exposure on olfaction: a review. Environ Health Perspect 124:1683–1693; http://dx.doi.org/10.1289/EHP136

## Introduction

Olfactory dysfunction is a significant public health problem worldwide, with a prevalence estimated to be between 13.9% and 31.7% in older adults (~ 60–90 years of age) in the United States (Murphy et al. 2002; Pinto et al. 2014a; Rawal et al. 2016; Schubert et al. 2012), with similar proportions reported in Sweden (Brämerson et al. 2004) and Germany (Landis et al. 2004; Vennemann et al. 2008). This sensory deficit is associated with decreased quality of life (Smeets et al. 2009), including an impaired ability to detect hazards such as gas leaks or fires (Santos et al. 2004) and decreased sex drive (Toller 1999), possibly driven by increased depression and distress (Gudziol et al. 2009; Smeets et al. 2009). Further magnifying the importance of this condition is the fact that olfactory loss presages major neurodegenerative conditions (Devanand et al. 2000; Ross et al. 2008) and death in older adults (Gopinath et al. 2012; Pinto et al. 2014b; Wilson et al. 2011). With the aging of the world’s population (Lutz et al. 2008), the prevalence of olfactory loss is likely to increase. Although the causes of olfactory loss in the general population are not well defined, it is possible that air pollution exposure may hasten olfactory loss, given its well-documented deleterious effects on health and the anatomic position of the olfactory apparatus that directly exposes it to the external environment.

Environmental effects on olfaction have long been suspected, especially in an industrial or occupational setting (Doty 2006; Gobba 2006). However, occupational exposures tend to involve relatively high concentrations of industrial toxins that are experienced only rarely by most members of the general population. Preliminary evidence suggests that air pollution exposures at levels experienced more commonly by the general population also impact olfaction in humans. For example, airborne fine particulate matter (PM_2.5_; aerodynamic diameter ≤ 2.5 μm) has been found to enter the olfactory epithelium, to be transported to the olfactory bulb, and to even reach the olfactory cortex and other brain regions (Calderón-Garcidueñas et al. 2003a). Despite the potential magnitude of the harmful impact on olfactory function, our understanding of this impact remains limited, because large-scale population data have not yet been analyzed for effects on olfaction as they have been on respiratory and cardiovascular disease. Here, we will review the existing data on the relationship between olfaction and ambient air pollution, with a focus on the evidence found in human epidemiologic and pathophysiologic studies. Our goals in conducting this review were to *a*) examine all relevant human data evaluating links between ambient air pollution exposure and olfactory function, *b*) examine all relevant human data with supporting animal data to examine potential mechanisms for air pollution-induced olfactory loss, and *c*) identify gaps in the literature as areas for future research.

## Methods

Utilizing the assistance of experienced, professional academic reference librarians, we performed a structured literature search focused on first identifying all relevant human studies related to olfaction and pollution. Multiple databases were queried on 3 August 2015: PubMed was searched by use of both MeSH term and keyword searches, and Web of Science was searched for additional studies using a keyword search. Papers were included if they were tagged with at least one search term from Group A and at least one search term from Group B (Table 1). As this search was limited to human studies, papers found in PubMed were restricted based on MeSH terms to exclude all studies tagged with “animals,” but to include studies tagged with both “animals” and “humans.” PubMed papers not yet indexed in MEDLINE and those found in Web of Science were examined manually to include only those with a focus on humans.

**Table 1:** Search terms.

Search terms and databases	Group A terms	Group B terms
MeSH terms PubMed only	Inhalation exposure	Olfaction disorders
	Air pollution	Smell
	Air pollutants	Olfactory nerve diseases
		Olfactory pathways
		Olfactory nerve
Keywords PubMed and Web of Science	Pollut*	Olfact*
	“Environmental exposure”^†^	Smell
		“Odor identification”^†^
		“Odor threshold”^†^
		“Odor discrimination”^†^
*End-truncated search terms. ^†^Terms entered into database with quotation marks to return results with exact matches.		

Papers were excluded if they were not written in English, as were case studies, reviews, editorials, other non-original research articles, or those that did not examine some facet of the association between environmental pollution and olfaction. Epidemiologic studies were restricted to those with an objective measure of olfactory function (i.e., odor identification, threshold, or discrimination, but not self-reported olfaction, “odor nuisance,” or “odor annoyance”). Studies examining any type of occupational exposure were also excluded, as were studies related to tobacco smoke exposure that did not examine ambient air pollution. Human pathophysiologic studies were limited to those evaluating ambient air pollutants and specifically examining olfactory epithelium (or nasal cavity regions known to contain olfactory epithelium) or olfactory bulb tissue, rather than nasal mucosa, which may contain both respiratory and olfactory epithelia.

After reviewing these articles, we then performed parallel search strategies for animal studies, including terms related to chronic exposures to exclude high-dose single exposure studies and more realistically target data on exposure to ambient air pollutants identified by the first search. A search of animal studies was restricted to topics for which relevant human studies had been found, to focus on possible pathophysiologic mechanisms relevant to humans.

Using this search strategy, we identified a total of 878 unique articles in English: 560 in PubMed and 445 in Web of Science. After other exclusion criteria were accounted for, 17 articles remained for review. The bibliographies of these 17 papers were also examined for additional relevant articles, adding 1 additional article beyond those found in the primary database searches. A total of 18 articles were thus identified; they are summarized in Table 2 (epidemiologic) and Table 3 (pathophysiologic). A post hoc review of abstracts from non-English papers, where available, did not yield any potentially relevant articles that were excluded from this review. Parallel strategies were used to identify animal studies to support human evidence and are discussed separately from human studies. We begin first with brief overviews of relevant olfactory anatomy, olfactory function testing, and olfactory system disorders and their causes, before discussing evidence from the literature search linking pollution exposure and olfaction in a narrative review.

**Table 2:** Summary of human epidemiologic studies of pollution and olfaction.

Epidemiologic studies	Study population	Olfaction measure	Pollution measure	Results
Hudson et al. 2006	Convenience sample of Mexico City, Mexico residents (*n *= 82); Tlaxcala, Mexico residents (*n *= 86).	Odor ID and threshold using coffee and orange drink. Odor discrimination using horchata and atole.	Ambient air pollution. Mexico City residents assumed to have higher exposure than Tlaxcala residents.	Tlaxcala residents outperformed Mexico City residents at all three tasks. Differences limited to ages 20–49 years, but not in 50–63 years.
Guarneros et al. 2009	Convenience sample of Mexico City, Mexico residents (*n *= 30); Tlaxcala, Mexico residents (*n *= 30).	Sniffin’ Sticks: odor ID, threshold, and discrimination.	Ambient air pollution. Mexico City residents assumed to have higher exposure than Tlaxcala residents.	Tlaxcala residents significantly outperformed Mexico City residents in threshold, discrimination, and sum of all 3 olfactory domains, but not in odor ID.
Calderón-Garcidueñas et al. 2010 (also a pathophysiologic study)	Convenience sample of Mexico City, Mexico residents (high exposure, *n *= 62); Polotitlán, Mexico residents (low exposure, *n *= 25).	UPSIT	Ambient air pollution. Mexico City residents assumed to have higher exposure than Polotitlán residents.	Polotitlán residents outperformed Mexico City residents.
Sorokowska et al. 2013	Convenience sample of Germany residents (*n *= 286); Bolivian rainforest Tsimane’ people (*n *= 151).	Olfactory threshold testing.	Ambient air pollution. Germany residents assumed to have higher exposure than Tsimane’.	Tsimane’ outperformed Germany residents.
Sorokowska et al. 2015	Convenience sample of Wroclaw, Poland residents (*n *= 168); Bolivian rainforest Tsimane’ people (*n *= 151); Cook Islands residents (*n *= 61).	Olfactory threshold testing.	Ambient air pollution. Poland residents assumed to have higher exposure than Tsimane’ and Cook Islands residents.	Poland considered high exposure because it is an industrialized nation. Cook Islands residents outperformed residents of Poland and Tismane’. Tsimane’ outperformed residents of Poland.
Ranft et al. 2009	Older German women 68–79 years old (*n *= 377), subset of Study on the Influence of Air Pollution on Lung Function, Inflammation, and Aging (SALIA).	Sniffin’ Sticks: odor ID.	PM_10_ at nearest pollution monitoring site. Motor vehicle exhaust measured as distance to nearest busy expressway.	PM_10_ not associated with olfaction. Motor vehicle exposure associated with significantly poorer olfaction in multivariate analysis.
Prah and Benignus 1979	Convenience sample of male subjects (*n *= 8).	Olfactory threshold for butyl alcohol and acetic acid.	Exposed to 400 ppb O_3_ for 4 hr/day for 4 days.	Initially increased olfactory threshold (worsened olfaction), but this effect diminished by 3rd day of exposure.
Lucchini et al. 2012	Convenience sample of children 11–14 years old in Valcomonica, Italy (area of ferroalloy plants, *n *= 154) and Garda Lake, Italy (control location, *n *= 157).	Sniffin’ Sticks: odor ID.	Mn measured in air (personal samplers), soil, tap water, and in participant hair, blood, and urine.	Soil Mn associated with poorer olfaction, but airborne Mn and internal Mn not associated with olfaction.
Lucchini et al. 2014	Convenience sample of elderly subjects 65–75 years old in Valcamonica, Italy (area of ferroalloy plants) and control locations (*n *= 255).	Sniffin’ Sticks: odor ID.	Mn measured in air (personal samplers), soil, tap water, and in participant blood and urine.	Soil and airborne Mn associated with poorer olfaction.
Guarneros et al. 2013	Convenience sample of residents near a mining district in Hidalgo, Mexico (*n *= 30); residents of Calnali, Mexico (*n *= 30).	Sniffin’ Sticks: odor ID, threshold, and discrimination.	Mn. Hidalgo residents had higher exposure than Calnali residents based on hair samples.	Calnali residents significantly outperformed Hidalgo residents in all three olfactory domains. However, no dose–response between hair Mn and olfaction.
Grashow et al. 2015	Elderly men near Boston, USA (*n *= 165), from the Normative Aging Study (NAS).	UPSIT, given an average of 12 years after bone Pb measurement.	Pb measured in patella (cumulative 8–10 year exposure) and midtibial shaft (cumulative exposure over decades).	Tibial Pb significantly associated with poorer olfaction. Patella Pb associated with decreased UPSIT score, but this was nonsignificant.
Broder et al. 1988a	Survey study near Toronto, Canada of residents living in homes insulated with urea formaldehyde foam (*n *= 1,726); residents living in control homes (*n *= 720).	Odor threshold for pyridine.	Formaldehyde, found to be higher in exposed homes than in control homes.	No significant differences in olfactory function between residents of exposed homes and control homes.
Fitzgerald et al. 2008	Survey of older adults 55–74 years old from Fort Edward, Hudson Falls, and Glens Falls, New York, USA (*n *= 253).	UPSIT	PCBs measured in blood.	No association between serum PCB and olfactory function.
Fitzgerald et al. 2012	Survey of older adults 55–74 years old from Fort Edward, Hudson Falls, and Glens Falls, New York, USA (*n *= 144).	UPSIT	PBDEs measured in blood.	No association between serum PBDE and olfactory function.
ID, identification; Mn, manganese; O_3_, ozone; Pb, lead; PBDEs, polybrominated diphenyl ethers; PCBs, polychlorinated biphenyls; ppb, parts per billion; UPSIT, University of Pennsylvania Smell Identification Test.				

**Table 3:** Summary of human pathophysiologic studies of pollution and olfaction.

Pathophysiology Studies	Participants	Tissue analyzed	Pollution exposure	Results
Calderón-Garcidueñas et al. 1998	Young adults 19–35 years old from southwest Mexico City (*n *= 54) and Isla Mujeres in Caribbean (*n *= 12).	Nasal biopsies: middle turbinate (representative of olfactory epithelium).	Ambient air pollution, particularly O_3_. Mexico City residents assumed to be higher exposed than Isla Mujeres residents.	Mexico City residents’ biopsies showed basal cell hyperplasia, squamous metaplasia, and epithelial dysplasia. Isla Mujeres residents had normal biopsies.
Calderón-Garcidueñas et al. 2004	Residents of Mexico City and Monterrey (*n *= 3). Residents of Abasolo, Iguala, El Mante, Tlaxcala, or Veracruz (*n *= 2).	Brain tissue: olfactory bulbs and olfactory nerves.	Ambient air pollution. Mexico City and Monterrey residents assumed to be higher exposed than Abasolo, Iguala, El Mante, Tlaxcala, and Veracruz residents.	Olfactory bulbs from all 3 Mexico City and Monterrey residents contained COX-2 and beta-amyloid, and olfactory nerves contained beta-amyloid. Neither control city residents’ olfactory tissues contained COX-2 or beta-amyloid.
Calderón-Garcidueñas et al. 2008	Residents of Mexico City (*n *= 35). Residents of Tlaxcala or Veracruz (*n *= 12).	Brain tissue: olfactory bulbs and olfactory nerves.	Ambient air pollution. Mexico City residents assumed to be higher exposed than Tlaxcala and Veracruz residents.	Among Mexico City residents’ olfactory tissues, 4 contained PM, 6 contained beta-amyloid, and 3 contained α-synuclein, none of which were found in any control city residents’ brains. Mexico City residents’ olfactory tissues also had elevated COX-2, IL-1β, and CD14 compared to control city residents’ tissues.
Calderón-Garcidueñas et al. 2010 [also an epidemiologic study]	Mexico City, Mexico residents (high exposure, *n *= 35). Polotitlán, Mexico residents (low exposure, *n *= 9).	Brain tissue: olfactory bulbs and olfactory nerves.	Ambient air pollution. Mexico City residents assumed to be higher exposed than Polotitlán residents.	Among Mexico City residents’ olfactory tissues, 2 contained PM, 29 showed immunoreactivity to beta-amyloid, 5 contained beta-amyloid plaques and lipofuscin granules. Control city residents’ olfactory tissues were unremarkable.
Calderón-Garcidueñas et al. 2013	Residents of Mexico City (*n *= 47). Residents of Tlaxcala or Veracruz (*n *= 12).	Brain tissue: olfactory bulbs and olfactory nerves.	Ambient air pollution. Mexico City residents assumed to be higher exposed than Tlaxcala and Veracruz residents.	Mexico City residents’ olfactory bulbs had higher COX-2 and IL-1β. No differences in DNA repair enzymes (LIG1, OGG1, XPA) between Mexico City and control city residents. Higher levels of Ni, Mn, and Cr in frontal lobes of Mexico City residents than of control city residents.
Note: COX-2, cyclooxygenase-2; Cr, chromium; IL-1β, interleukin 1 beta; LIG1, DNA ligase I; Ni, nickel; OGG1, 8-oxoguanine DNA glycosylase; XPA, xeroderma pigmentosum group A complementing protein.				

## Olfactory Anatomy

**Figure 1:**
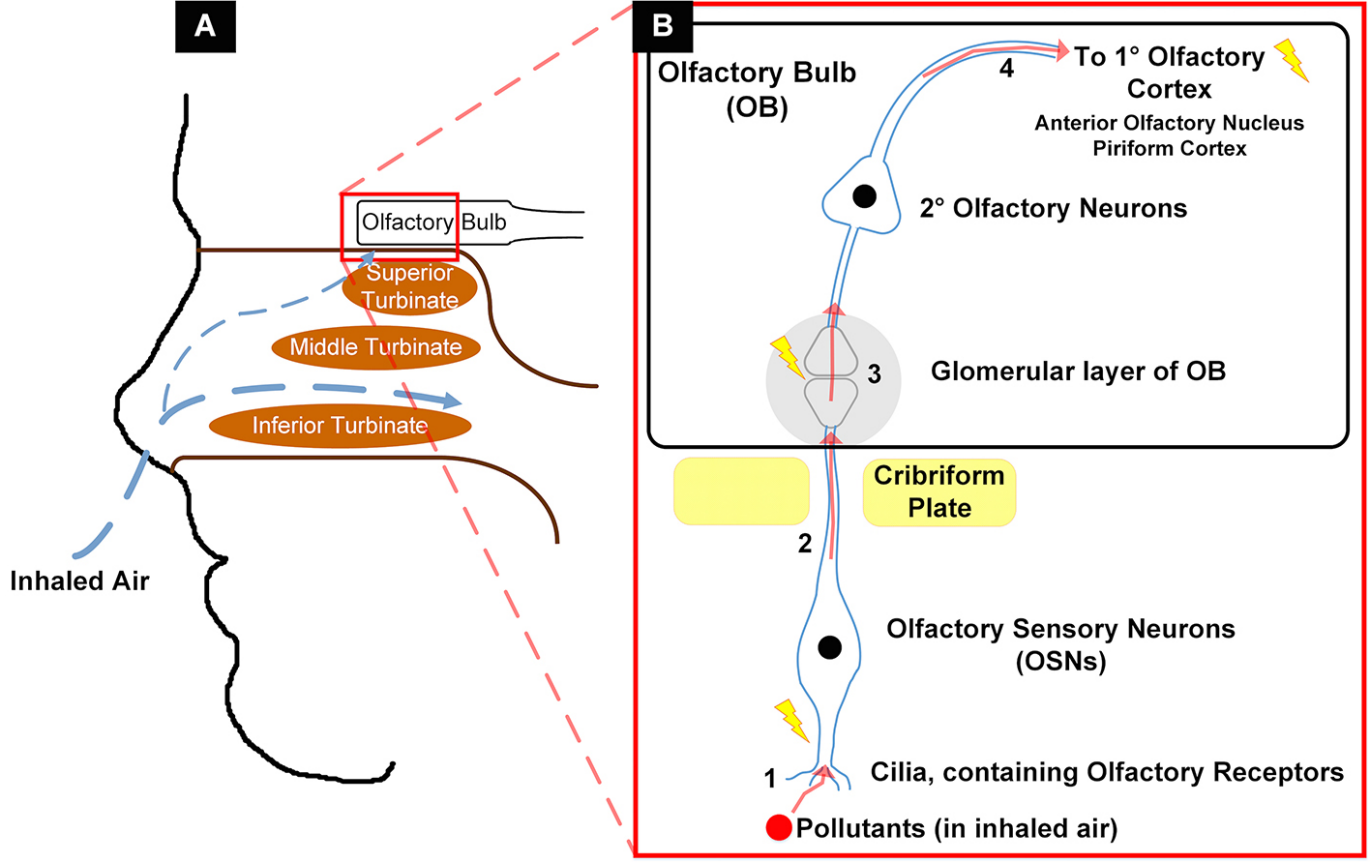
Summary of proposed mechanism of air pollution entry into olfactory tissues. (*A*) Passage of inhaled air through nasal cavities, including small airflow up to olfactory mucosa. (*B*) Passage of inhaled airborne pollutants through olfactory tissue: 1, uptake of pollutant by olfactory sensory neurons (OSNs); 2, translocation up OSNs’ axons (CN I) through cribriform plate to the olfactory bulb (OB); 3, uptake of pollutant within OB by 2° olfactory neurons; 4, translocation along 2° olfactory neurons to 1° olfactory cortex. Lightning bolts indicate sites of pollution-induced cellular stress, cytotoxicity, and inflammation.

The nose is usually the point through which air pollution enters the human body, placing it at risk for injury from toxins, pathogens, and injurious molecules in the inspired air. Of note, odorants in inspired air can also be perceived as irritants via the trigeminal nerve (CN V) in the nasal vestibule and cavity (Doty 2003), but in this article we limit our discussion to olfactory chemosensation via the first cranial nerve (CN I). A visual summary of relevant olfactory anatomy and these sites at which pollutants can impact olfactory function is presented in Figure 1. As described in more detail by Doty (2003), odorants travel into the body with inspired air, passing through the nose and through specific regions dedicated to olfaction (Figure 1A). As they pass through these regions, odorants can bind to olfactory sensory neurons (OSNs) in the olfactory epithelium (sometimes called the olfactory neuroepithelium because it contains olfactory neurons of CN I) (Figure 1B). Odorant binding to receptors on the surface of OSNs initiates signal transduction down their axons (making up CN I), which travel through foramina in the cribriform plate and synapse in the glomerular layer of the olfactory bulb, the first site of sensory input integration. Within the olfactory bulb there are a wide variety of synaptic connections that eventually project to the primary olfactory cortex, which is made up of components of several structures, including the anterior olfactory nucleus and the piriform cortex. From there, olfactory signals can project to a third site of signal organization, including the orbitofrontal and insular cortices, thalamus, hypothalamus, and amygdala. Through these same pathways, pollutants can enter the olfactory epithelium, be transported to the olfactory bulb, and even reach the olfactory cortex and other brain regions (Calderón-Garcidueñas et al. 2003a) (Figure 1). We note that pollutants may also impact olfaction through indirect effects on nasal mucosa that lead to overall sinus disease.

Of all inspired air, only a small fraction reaches the olfactory mucosa; thus, only a small percentage of inhaled odorants (or airborne pollutants) can reach olfactory tissue. This has been modeled by a wide variety of computational fluid dynamics (CFD) studies, with the olfactory region estimated to receive between 2% and 16% of airflow entering the nasal cavity (Hahn et al. 1993; Keyhani et al. 1995; Schroeter et al. 2008, 2010). Thus, a small, but significant proportion of air (and airborne pollutants) may reach the olfactory epithelium, be deposited, and initiate a host response.

## Olfactory Function Testing

Olfaction can be measured by a variety of methods, and examples of currently available validated tests are summarized in Table S1. Common aspects of olfaction that are typically measured include odor identification, odor threshold, and odor discrimination. Odor identification requires the participant to name correctly a presented odor, often from a list of choices presented as words or pictures. Odor threshold testing involves determining the lowest concentration at which a particular odor can be discerned correctly from (usually) two negative controls (with the examination taking place in non-odorous ambient air). In odor discrimination testing, a participant chooses which odorant of three is different from the other two. These tests can be considered to be relatively objective measures of olfactory ability, as contrasted from non-objective methods, such as self-reported olfactory dysfunction or odor annoyance score (Knaapila et al. 2008). These two subjective assessments have been found to be associated with each other, but not with objectively measured olfactory ability (Knaapila et al. 2008). Self-reported olfactory ability in particular has been found to be an unreliable metric when compared to measured olfactory function (Landis et al. 2003).

## Olfactory System Disorders and Causes

Olfactory loss has been described as reduced olfactory function (hyposmia) and total loss of function (anosmia). Loss of olfaction associated with aging is called presbyosmia, and age remains the strongest risk factor for this sensory impairment (Doty and Kamath 2014). Other causes of olfactory loss include viral upper respiratory infections, neurodegenerative conditions (e.g., Alzheimer’s disease or Parkinson’s disease), head trauma, congenital conditions (e.g., Kallmann’s syndrome), and toxic occupational exposures (Doty 2003).

Other olfactory system disorders include parosmia, in which an odorant elicits an incorrect, often foul, smell. Euosmia is a subset of parosmia that refers to patients experiencing a pleasant but incorrect smell upon presentation with an odorant. Another disorder, phantosmia, refers to the perception of smell in absence of an odorant. This phenomenon is also termed “olfactory hallucinations” and often associated with epilepsy, particularly with temporal lobe foci (Doty 2003).

## Epidemiology

We organized our review of epidemiologic studies into studies of general ambient air pollution, and then specific individual pollutants of mechanistic relevance to which the general population is exposed. Details of these studies, including study population, olfaction and pollution measures, and a brief summary of results are presented in Table 2. One experimental study in which human subjects were exposed to ozone is also included here.

### Ambient Air Pollution

Evidence of an association between occupational exposure to pollutants and loss of olfaction goes back several decades, but studies of air pollution exposures experienced by the general population and olfaction have emerged only in the past 10 years. The first epidemiologic work we found that examined ambient air pollution and olfaction was conducted in 2006 (Hudson et al. 2006). This study compared residents of Mexico City, which has notably high levels of air pollution, especially inhalable PM_10_ (particle matter with an aerodynamic diameter < 10 μm), PM_2.5_, and ozone (O_3_) (Calderón-Garcidueñas et al. 1997, 1998, 2001b), and residents of the nearby city of Tlaxcala, which reportedly has lower pollution levels. Given their proximity and shared culture, language, diet, and other factors that may confound any observed associations, comparisons of olfaction between residents of these similarly located Mexican cities may be more relevant than those made across countries (as in some other studies). Concentrations of PM and O_3_ in Mexico City are high relative to those found in the United States, with concentrations of PM_10_, PM_2.5_, and O_3_ reaching average levels around 78 μg/m^3^, 22 μg/m^3^, and 0.250 ppm, respectively (Calderón-Garcidueñas et al. 2001b; U.S. EPA 2016b). Concentrations in Mexico City routinely exceed National Ambient Air Quality Standards (NAAQS) set by the U.S. Environmental Protection Agency (EPA) (for PM_10_, PM_2.5_, and O_3_, which were 50 μg/m^3^, 15 μg/m^3^, and 0.08 ppm, respectively, between 1997–2006 (U.S. EPA 2016c, 2016d). Pollution concentrations in Tlaxcala, a control city with low pollution, were not reported in these studies, although authors stated that levels were below the U.S. NAAQS (Calderón-Garcidueñas et al. 2006; Hudson et al. 2006).

Residents of Mexico City and Tlaxcala were given odor identification and threshold testing tasks with instant coffee and orange drink used as odorants, as well as an odor discrimination test between two similar smelling local beverages, horchata and atole (Hudson et al. 2006). The residents of Tlaxcala (low pollution) detected coffee and orange drink at lower concentrations and were better able to discriminate between the two beverages. Interestingly, the differences were observed among participants 20–49 years old, with minimal or no differences among participants 50–63 years of age. This may indicate that the effects of pollution become manifest at young ages and are not readily apparent in older adults, who may have experienced substantial age-related olfactory decline. For the odor identification task, there were no differences between residents of the two cities in the concentration at which they could identify (i.e., name) the odorants.

Two subsequent studies have been conducted in similar populations by use of more standardized olfactory function testing protocols (Calderón-Garcidueñas et al. 2010; Guarneros et al. 2009) (Table 2). Guarneros et al. (2009) found that the residents of Tlaxcala with low pollution (control subjects) performed significantly better in olfactory function tests than did the subjects from Mexico City with high pollution, scoring a mean of ~ 37 on the aggregate score versus a mean of ~ 34 for those with high exposure (Table 2). When the differences across the three olfactory domains tested were evaluated, differences were observed in threshold and discrimination, but not identification, tasks; these findings were similar to the results from the previous study in these two cities (Hudson et al. 2006). Similar results were found in a 2010 study that compared residents from Mexico City to controls from Polotitlán, a nearby town with roughly 5% lower levels of PM_10_ and a slightly lower percentage of PM_2.5_ out of total PM_10_ (40% in Polotitlán vs. 46% in Mexico City) (Calderón-Garcidueñas et al. 2010). For other criteria air pollutants (CAPs), levels in Polotitlán were reported to fall within U.S. NAAQS (Calderón-Garcidueñas et al. 2010). Subjects from Mexico City were found to perform significantly worse on odor identification testing (Table 2). Interestingly, the mean age in this study was 21 years, another possible indication that pollution may impact olfaction in younger people who are likely to have intact olfactory systems prior to additional decrement from age-related decline. However, there were sex differences between the Mexico City and control study groups: 41 out of 62 Mexico City residents were male vs. 14 out of 25 Polotitlán residents (control city). As it is well established that males are poorer smellers than females (Brand and Millot 2001), it is possible that these results were in part confounded by sex.

These studies have consistently found impaired olfactory ability of residents living in Mexico City compared to nearby controls, even after basic demographic covariates were accounted for. Across the olfactory threshold and discrimination domains, considered purer tests of olfaction than odor identification (which has a strong cognitive component) (Larsson and Bäckman 1997), the residents of control cities consistently outperformed residents of Mexico City. However, a significant difference in odor identification was seen only in one of three studies (Calderón-Garcidueñas et al. 2010). Confounding factors such as cognitive function cannot, of course, be entirely excluded. Additionally, dose–response relationships cannot be studied given the binary nature of the exposure (air pollution) estimate, and one cannot exclude the fact that other unobserved differences may explain the association. Such differences may include genetic variation between these groups, differences in olfaction utilization between individuals living in cities with differing degrees of urbanization, or some cultural differences. However, given that the cities studied were all within the same region of Mexico, genetic and cultural variations are unlikely to be drastically different.

Two recent studies have used similar methods to compare residents of industrialized nations to residents of non-industrialized regions who were hypothesized to face lower pollution exposures (Sorokowska et al. 2013; Sorokowska et al. 2015). To compare the olfactory ability of participants with high- and low-pollution exposures, Sorokowska et al. (2013) compared subjects from industrialized Germany to those from the Bolivian rainforest (Table 2). The authors found that the Tsimane’ people of the Bolivian rainforest had significantly lower olfactory thresholds (i.e., better olfactory function) than did Germans. In a follow-up study, Sorokowska et al. (2015) compared the odor threshold among subjects from the non-industrialized Cook Islands in the South Pacific to that of subjects from industrialized Wroclaw, Poland, and the Tsimane’ of the Amazon (Table 2). The subjects from the Cook Islands had olfactory performance that was significantly superior to that of subjects from Poland and the Tsimane’. This difference was not explained by differences in smoking, but may have been partly due to higher alcohol use among Polish subjects, who also performed worse than the Tsimane’ on olfactory testing. Although it is possible that country specific differences in olfactory ability are due to higher air pollutant exposures experienced by residents of industrialized countries, these differences could also be explained by numerous other factors, including a greater need for olfactory ability among non-industrialized people who rely on farming and hunting and gathering for subsistence, cultural and genetic variations between these two very different groups, health differences, and large expected differences in early life exposures and life-course experiences. Nonetheless, findings from these country comparisons are consistent with the work in Mexico and support the concept that pollution may have deleterious effects on olfaction in humans.

In a German study of older women who had lived at their current address for at least 20 consecutive years, Ranft et al. (2009) found a significant dose response between olfaction and exposure to vehicle exhaust, a key source of multiple air pollutants, including the CAPs PM_2.5_, carbon monoxide (CO), and nitrogen dioxide (NO_2_) (Brugge et al. 2007). In a multivariate analysis, this association was stronger than all measured covariates other than depression. There was no observed effect of PM_10_, measured at the pollution monitoring station nearest to each participant’s home. An important limitation of this study, however, is that vehicle exhaust exposure was estimated based on the distance between the participant’s residence and the nearest busy expressway (defined as having > 10,000 cars per day), an indirect measure of motor vehicle exposure. This study included participants from a variety of environments (urban to rural), allowing for variation in participant exposure to air pollution.

These epidemiologic studies have generally used well-validated tests of olfactory function but have several significant limitations. The majority of the work has relied on proxy measures of air pollution exposure, through comparisons of residents of different cities or different countries. Further, they are of generally small, restricted sample sizes, with no broad epidemiologic study of a population to date. Many of these studies, despite their pioneering aspects, have failed to account appropriately for important potential confounders.

### Individual Pollutants

***Ozone (O_3_).*** O_3_, a secondary pollutant, was shown experimentally to transiently worsen olfaction in a group of eight males exposed to extremely high levels of O_3_ for 4 days (Prah and Benignus 1979). However, this effect had diminished by the third day of exposure. The rapid diminishment of the effect of O_3_ may be related in part to associated increases in expression of endogenous antioxidants such as superoxide dismutase (SOD). This was found to be the case in a study of acute (4 hr) O_3_ exposure ranging from 0.1 to 1 ppm and memory formation in rats, where animals exposed to low doses of ozone (0.2 ppm) had the greatest increases in SOD expression and the greatest long-term memory retention as well (Rivas-Arancibia et al. 1998). Prah and Benignus also acknowledged the need for further studies, given the very small sample size in this study and its high ozone exposures, which were higher than would be experienced by populations living in the US. Given the known adverse health effects of ozone (Bell et al. 2014; Medina-Ramón et al. 2006; Nuvolone et al. 2013), future studies in humans would need to be performed with lower exposures.

***Lead (Pb).*** Cumulative exposure to Pb, a heavy metal and also one of the CAPs (U.S. EPA 2016b), has also recently been found to correlate with poorer olfactory function among a cohort of older men (Grashow et al. 2015). Pb is primarily stored in bone (Barry 1975), thus bone measurements were compared to olfactory function test results performed an average of 12 years later. Patellar Pb is considered a biomarker of 8–10 years of cumulative exposure, and tibial Pb is a marker of exposure long term (decades) (Wilker et al. 2011). The authors found that increased tibial Pb was associated with poorer olfaction after controlling for age and current smoking. While no significant association was seen with patellar Pb, the exposure–olfaction association trended in the same direction as tibial Pb. Inhaled Pb is likely able to directly reach olfactory tissues and translocate to the brain, given its strong deleterious neurocognitive effects. However, this study was unable to assess precisely the timing of exposures and whether the majority of exposure came via inhalation versus ingestion.

***Manganese (Mn).*** There is also evidence that exposure to another heavy metal, Mn, may adversely impact olfaction. Mn is a naturally occurring pollutant that is also released from various industrial sources and may be found in unleaded gasoline (U.S. EPA 2015, 2016a). Indeed, in Canada, sites with high-vehicle traffic have been found to have significantly higher levels of airborne Mn—higher than the inhalation reference concentration (0.05 μg/m^3^) published by the U.S. EPA Integrated Risk Information System (IRIS)—than did low-traffic sites (Boudia et al. 2006; U.S. EPA 1993). In Italy, a study of exposure to Mn and olfactory function in children showed that olfactory function was inversely associated with Mn concentrations in soil, but not airborne Mn or internal Mn (based on an analysis of participants’ urine, blood, and hair) (Lucchini et al. 2012). The authors asserted that this was due to the fact that the last ferroalloy plants had closed several years prior to the study, and therefore soil Mn would be a better indicator of the participants’ exposure level during the period when the plants were functioning; airborne exposure and biomarkers may instead be reflecting current exposure, which may not be as relevant for olfaction. Soil Mn concentrations may even be reflective of historic airborne Mn exposure, the most relevant exposure route, as airborne Mn may be driven by suspension of soil Mn into the air (Boudissa et al. 2006). However, the presumed effects of Mn may still be driven by inhalation exposure, as it is inhaled Mn that may directly reach olfactory tissues (Aschner et al. 2005).

A follow-up study in Italy by Lucchini et al. (2014) examined odor discrimination ability among elderly subjects living in an area were ferroalloy plants were previously located. They found that both soil and airborne Mn were significant independent predictors of poorer olfaction, with a significant interaction between these two exposures. These two studies in Italy are limited by their narrow geographic scope and in that the age groups examined were those particularly susceptible to toxic exposures, namely children and the elderly (Lucchini et al. 2012, 2014). However, they do demonstrate a consistent trend of higher Mn exposure being related to poorer olfaction. Interestingly, in neither study was internal Mn significantly associated with olfaction. This is perhaps an indication that exposure to olfactory tissues is most relevant for an ultimate impact on olfaction, irrespective of absorption in the bloodstream. Alternatively, it is also possible that environmental Mn acts as a surrogate for other chemicals or pollutants, as industrial releases are likely to be a complex mixture of many potentially harmful substances.

Similarly, Guarneros et al. (2013) found that people living close to a Mn mining district in Mexico performed significantly worse in olfactory testing compared to a non-exposed group of people. This also suggests that Mn exposure is associated with olfactory dysfunction, but in this study no dose–response association was observed between Mn concentrations in the hair and olfactory performance of the participants.

***Other pollutants.*** A few other pollutants have been assessed for an association with olfactory function. Formaldehyde is an ambient air pollutant that is also an important indoor exposure for people living in homes insulated with formaldehyde-containing foam (Salthammer 2013). Residing in homes with this insulation was not found to be correlated with poorer olfaction, compared to residing in formaldehyde-free homes (Broder et al. 1988a, 1988b). Although polychlorinated biphenyls (PCBs) and polybrominated diphenyl ethers (PBDEs) have been associated with a variety of adverse health outcomes (Bergkvist et al. 2016; Linares et al. 2015; Ruder et al. 2014), exposure levels as measured by serum concentration have not been found to be associated with olfactory function among older adults (Fitzgerald et al. 2008, 2012). However, the authors in these studies were unable to distinguish between routes of exposure to these compounds, and participants may not have faced primarily airborne exposures.

## Pathophysiology

Several studies have examined pathophysiological mechanisms behind air pollution-induced olfactory injury (Table 3). Much of the work directly examining olfactory tissue has been conducted in Mexico, where pathologic analysis of olfactory tissue from Mexico City residents has been compared to that of tissue from residents of cities or towns exposed to comparatively lower levels of urban pollution, particularly PM_2.5_ and O_3_ (Calderón-Garcidueñas et al. 2003b). Notably, air pollution levels are not regularly monitored in these smaller cities used as controls the way it is in Mexico City; thus, control cities are assumed to have lower levels of urban pollution based on a comparative lack of industry/motor vehicles and differences in geography. Studies of humans and dogs living in control cities have demonstrated a lack of air pollution-associated pathology in these subjects (Calderón-Garcidueñas et al. 2003b).

Calderón-Garcidueñas et al. (1998)found that nasal biopsies from young adults living in Mexico City exhibited pathologic changes compared to residents of a control town exposed to low air pollution (Table 3). Biopsies from the Mexico City residents showed basal cell hyperplasia, squamous metaplasia, and epithelial dysplasia in the anterior portion of the middle turbinate, a region chosen by the authors to represent exposure of the olfactory epithelium because it lies adjacent to air streams that reach the olfactory mucosa and that contains olfactory epithelium (Leopold et al. 2000). These participants also complained of upper respiratory symptoms such as nasal mucus, crusting, dryness, and obstruction, as well as epistaxis and rhinorrhea. In contrast, residents of the control town had no upper-respiratory symptoms and had normal biopsy results. These results suggest that airborne pollutants may directly damage the olfactory epithelium by inducing DNA damage (i.e., resulting in metaplasia), and ultimately cellular damage. Furthermore, the impact of exposure to air pollution can be observed histologically even in young adults, with associated upper-respiratory symptoms. This highlights an important consideration that ambient pollution exposure may cause sinonasal disease broadly and impact olfaction indirectly via pro-inflammatory effects on the sinonasal mucosa. Nasal obstruction and inflammation have been implicated in olfactory impairment, and there is evidence that anti-inflammatory treatment, such as corticosteroids, may improve olfaction in these patients (Wolfensberger and Hummel 2002).

Calderón-Garcidueñas et al. (2004, 2008, 2010, 2013) have also found pathologic evidence in brain tissue, including the olfactory bulbs, of residents from Mexican cities with high pollution compared to those from cities with low pollution. A 2004 study showed that olfactory bulbs from three high-pollution residents contained cyclooxygenase-2 (COX-2) and beta-amyloid, in addition to beta-amyloid in supporting cells of the olfactory nerve, while none of the olfactory tissue from low-pollution residents contained COX-2 or beta-amyloid (Table 3) (Calderón-Garcidueñas et al. 2004). In 2008, an in-depth follow-up autopsy study of 35 Mexico City residents showed detectable particulate matter in neurons from either the glomerular region of the olfactory bulb or the olfactory nerve in four brains, whereas no particulate matter was found in brains of 12 control city residents (Table 3) (Calderón-Garcidueñas et al. 2008). Six olfactory bulbs from Mexico City residents contained beta-amyloid and three contained α-synuclein, compared to no olfactory bulbs from control city residents containing either protein. Significantly elevated levels of various inflammatory markers, COX-2, IL-1β, and CD14, were also found in olfactory bulbs from subjects in Mexico City, compared to levels in controls. The same research group found similar results in a later study, with evidence of particle accumulation, beta-amyloid plaques, and lipofuscin granules in olfactory nerve and olfactory bulb tissues of Mexico City residents, in addition to histologically abnormal layering and disorganization of the olfactory bulb (Calderón-Garcidueñas et al. 2010). In contrast, the brains from control residents were all unremarkable, with normally layered olfactory bulbs.

Another follow-up autopsy study of Mexico City residents and controls from lower-polluted cities showed significantly higher levels of COX-2 and non-significantly higher IL-1β in Mexico City residents’ olfactory bulbs (Table 3) (Calderón-Garcidueñas et al. 2013). On the other hand, the levels of three DNA repair enzymes, DNA ligase I (LIG1), 8-oxoguanine DNA glycosylase (OGG1), and xeroderma pigmentosum group A complementing protein (XPA), were similar in Mexico City and control residents’ olfactory bulbs, with no significant differences. This may have been due to significantly higher concentrations of heavy metals [nickel (Ni), Mn, and chromium (Cr)] in the brains of Mexico City residents. Notably, although the differences were not significant, concentrations of all other metals measured [vanadium (V), Pb, arsenic (As), zinc (Zn), selenium (Se), copper (Cu), cobalt (Co), and iron (Fe)] were higher in brains of Mexico City residents compared to controls as well. While the metal concentrations were measured only in the frontal lobes and not directly in olfactory bulb tissue, there were some significant correlations between metal concentrations in frontal lobe and gene expression in olfactory bulb tissue. Ni levels were correlated positively with IL-1β, and Cr and As were correlated positively with XPA. Interestingly, Zn, Co, and Cu levels were negatively correlated with DNA repair enzymes, and Se was negatively correlated with COX-2.

Taken together, evidence to date suggests that fine particles are able to accumulate in the olfactory bulbs of persons exposed to high levels of air pollutants, including particulate matter and heavy metals, potentially leading to local inflammation and neuropathology. Ozone may also play a role via a similar mechanism of inducing local inflammation (Kopp et al. 1999), though the effects of individual pollutants is difficult to distinguish given co-exposures. Notably, this group of studies is based on findings from autopsy studies of individuals who died spontaneously, calling into question the applicability of the results to the general healthy population. The highly exposed subjects in these studies have almost exclusively been from residents of a single urban location, Mexico City. Individuals from this particular city have also been treated as all having the same level of exposure, although this is unlikely to be the case (Alfaro-Moreno et al. 2002; Chow et al. 2002; O’Neill et al. 2004). Nonetheless, the results paint a consistent picture: the differences in brain and olfactory pathophysiology between highly exposed individuals and controls living in cities with lower levels of air pollutants, such as PM_2.5_ and O_3_, were substantial, as control brains rarely showed any pathology. Furthermore, the abnormal pathology in Mexico City residents was seen among children and young adults, indicating that pathologic damage can occur after a relatively small number of years of exposure.

## Animal Studies

The pathophysiological changes in human olfactory tissues related to pollution that were reported in these studies have also been identified in animal research. An examination of dogs from southwest Mexico City and Tlaxcala found that older dogs (age > 3 months) from Mexico City showed gross discoloration of the olfactory mucosa compared to that of younger dogs in Mexico City and dogs of all ages in Tlaxcala (Calderón-Garcidueñas et al. 2002), consistent with gross examination of the nasal mucosa of children living in this part of Mexico City (Calderón-Garcidueñas et al. 2001a). Histologically, the older dogs from Mexico City also showed degradation and disorganization of the olfactory epithelium, and consequently a thinner and disoriented epithelium. There were also histologic findings of metaplasia, a lack of nerve bundles and Bowman’s glands, and evidence of inflammation. However, the olfactory bulbs themselves showed largely unremarkable differences between high-exposure and control dogs. Most of these histological findings are consistent with nasal biopsies taken from children living in this highly polluted region of Mexico City (Calderón-Garcidueñas et al. 2001b), suggesting the ability of airborne pollutants to damage directly the olfactory epithelium. Another study of dogs living in southwest Mexico City showed that, in comparison to controls from Tlaxcala, they had elevated levels of inflammatory and cell stress markers in olfactory bulb tissue and evidence of increased DNA damage (Calderón-Garcidueñas et al. 2003a), consistent with results shown in human pathophysiology studies. Mice chronically exposed to O_3_ experienced atrophy of their olfactory epithelium, in addition to various other morphologic abnormalities to their nasal cavities (Herbert et al. 1996). Additionally, olfactory bulbs of mice exposed intranasally to 14 nm carbon black particles, fine particles used and produced in many manufacturing processes, for 4 weeks exhibit elevated mRNA levels for various markers associated with inflammation, including IL-1β, tumor necrosis factor alpha (TNF-α), C-C motif chemokine ligand 2 (CCL2), C-C motif chemokine ligand 3 (CCL3), and C-X-C motif chemokine ligand 9 (CXCL9), compared to controls (Tin-Tin-Win-Shwe et al. 2006). Notably, mice exposed to 95 nm carbon-black particles had no elevation of any of these markers, compared to controls, in olfactory bulb tissue. Mice in both exposure groups exhibited no such elevation of these markers in the hippocampal tissue. Although carbon black (not to be confused with black carbon, which is a heterogeneous component of ambient air pollution) exposure is mostly occupational (Long et al. 2013), it functionally illustrates here the ability of intranasal particle exposure to induce inflammation in the olfactory bulb. Furthermore, it demonstrates that this ability may be dependent on particle size.

Calderón-Garcidueñas et al. (2003a) also showed that Ni and V concentrations were highest in the olfactory mucosa of dogs and were progressively lower in the olfactory bulb and the frontal cortex. This may indicate that airborne pollutants can undergo retrograde transport and travel from the site of initial exposure (the olfactory epithelium) to the brain. Thus, inhaled pollutants may cause direct damage to the olfactory epithelium and also olfactory nervous tissue through retrograde translocation to the olfactory bulb from olfactory mucosa. This is indeed the case, and has been found consistently with Mn in particular.

Tjälve et al. (1995) demonstrated that Mn exposed to olfactory regions of pike was taken up by olfactory receptors and transported to the olfactory bulb, where it accumulated, but was also able to undergo further transport from the olfactory bulb to other brain structures derived from the telencephalon and diencephalon. However, not all heavy metals are able to undergo such transport. For example, cadmium (Cd), which is associated with olfactory dysfunction (Gobba 2006), has been found to not undergo further retrograde transport beyond the olfactory bulb (Tjälve et al. 1996; Tjälve and Henriksson 1999). This may indicate that transport beyond the olfactory bulb to the olfactory cortex is not necessary to disrupt olfactory function. Similar to findings in the previously discussed studies, the ability of heavy metal particles to travel via this pathway is size-dependent: Mn particles 1.3 μm in diameter were able to undergo some transport to the olfactory bulb but, particles 18 μm in diameter did not lead to any Mn accumulation in the olfactory bulb (Fechter et al. 2002). Size-selection mechanisms may be at the level of the olfactory receptors in the olfactory epithelium or occur during axonal transport to the olfactory bulb. Further, Mn particles < 100 nm in diameter are able to reach the olfactory bulb from the nose in rats and induce inflammation (Elder et al. 2006). Ultrafine particles (< 100 nm diameter) made of carbon are also able to undergo such retrograde transport to the olfactory bulb and possibly farther to other regions of the cerebrum (Oberdörster et al. 2004). This has been well documented in several follow-up studies as well (Nakane 2012), indicating that most inhaled particles of such a size are able at least to undergo translocation to the olfactory bulb via olfactory neurons, and may often, although not necessarily, be transported farther to the olfactory cortex and other brain regions.

Air pollutants have also been found to induce inflammation and cytotoxicity at the level of neuronal tissues, such as the olfactory bulb. For example, Mn exposure in mice was found to induce nuclear and organelle damage, necrosis, and later apoptosis in granule cells of the olfactory bulb (Colin-Barenque et al. 2011). The plasma membranes of these cells were also disrupted and disorganized, possibly from generation of Mn-induced reactive oxygen species generation that disrupts the normal membrane and cytoskeleton structure (Colin-Barenque et al. 2011). Diesel exhaust rich in nanoparticles (< 100 nm diameter) was also able to induce increased expression of N-methyl-D-aspartate (NMDA) receptor subunits and extracellular concentrations of glutamate, both of which may lead to excitotoxicity (Tin-Tin-Win-Shwe et al. 2009). Exposure to PM of various sizes is also associated with elevated expression of the antioxidant heme oxygenase 1 (HO-1) in the olfactory bulb, as well as in other brain tissues (Guerra et al. 2013). More generally, mice exposed to air in Mexico City, which is high in ambient air pollution, had elevated levels of IL-1β and CD14, which were associated with inflammatory responses, in their olfactory bulbs after 16 months of exposure, compared to controls breathing filtered air (Villarreal-Calderon et al. 2010). The neurotoxic effects of inhaled Mn can also be linked all the way to the olfactory cortex. Manganese sulfate inhalation in rhesus monkeys was found to lead to depressed levels of glutamate transporter-1 and glutamate/aspartate transporter in the olfactory cortex, two glutamate transporters in astrocytes necessary for recycling of glutamate to prevent excitotoxicity (Erikson et al. 2007).

Collectively, these findings show that ambient air pollutants can induce cytotoxic and inflammatory changes in the olfactory bulb and even in the olfactory cortex, possible sites for physiologic impairment leading to olfactory dysfunction. Chronic O_3_ exposure has been shown to reduce olfactory function in female rats (Guevara-Guzmán et al. 2009). These rats had indications of oxidative stress in their olfactory bulbs as well, tying the pathophysiologic damage in the olfactory tissue to phenotypic impaired olfactory function.

## Areas for Further Research

Further research is needed to clarify our understanding of the relationship between airborne pollutants and olfactory function. Much of the epidemiologic work to date has been limited to studies comparing a highly exposed group to a less-exposed group. However, this does not allow for robust examination of the pollution-olfaction relationship, such as dose–response trends, the impact of individual pollutants, cumulative effects, or the ability to control for confounders such as variations in pollution exposure or resident characteristics within or between cities and regions.

Thus, new larger-scale studies are required. These studies should include diverse populations, including racial and ethnic minorities, as they face greater burdens of air pollution exposures (Gray et al. 2013; Marshall 2008). As exposures hitherto have been largely estimated and reflective of recent exposures, there is also a need for studies considering personal pollution monitoring data and studies with the ability to evaluate the impact of cumulative lifetime exposures to these pollutants. Similarly, longitudinal studies are needed which assess the impact of childhood and early adulthood exposures on risk for future development of olfactory dysfunction, given the studies showing morphologic and molecular abnormalities in exposed children’s olfactory tissues. Given that impaired olfactory function may result from pollution exposure via an inflammatory pathway (e.g., by causing sinonasal disease), additional research is needed to evaluate the role that indirect effects of pollution on sinonasal mucosa may have in diminished olfaction. It is possible that pollution may impact olfaction via nasal congestion impeding odorant access to the olfactory cleft in addition to direct pathologic effects to the olfactory mucosa itself; however, no human or animal studies have to date been conducted to examine this possibility. Differential effects via acute and chronic exposure are also possible, but also have not been studied. Finally, given some evidence that genetic variation may lead to differential responses to air pollutants (Alexis et al. 2013; Calderón-Garcidueñas et al. 2015), there is a need to consider the impact of genotype on susceptibility to pollution-induced damage to olfactory tissue. Together, this additional information could help clarify the magnitude of pollution-associated anosmia, identify particularly susceptible groups, and point to areas for potential therapeutic intervention and/or prevention.

Additional research is also needed for a better understanding of the mechanisms that underlie pollution-associated olfactory dysfunction. This may also aid in providing an understanding of vulnerable populations and lead to the development of more effective therapeutic strategies for use in anosmic patients. To date, most of the work examining damage by pollutants on the nasal mucosa has focused on respiratory epithelium, with significantly less work on examining the olfactory epithelium. With complex heterogeneous air pollutants such as PM, it will be important to evaluate which characteristics and/or constituents are most relevant to damage of olfactory tissues. Additional studies must also be able to link pathophysiologic damage with measured olfactory function (i.e., phenotype). This may include patients with self-reported olfactory impairment; in fact, one recent study demonstrated an increased risk of self-reported impairment associated with self-reported air pollution exposure, among the general population in Korea (Lee et al. 2013). However, the accuracy of self-reported data remains unclear (Hoffman et al. 2009; Knaapila et al. 2008; Landis et al. 2003; Rawal et al. 2014) and merits additional study given the potential uses for a patient-reported phenotype. Such research may also improve our understanding of the association between pollution exposure and risk for conditions associated with olfactory dysfunction, such as neurodegenerative diseases.

## Conclusions

In summary, we have identified and reviewed human studies that examined ambient air pollution and olfaction. The data from these studies, taken together with results from relevant animal studies, show evidence that ambient pollution exposure may be linked with impaired olfaction. These toxins can directly damage the olfactory mucosa upon inhalation, through direct cytotoxic effects and DNA damage, often resulting in squamous metaplasia of this tissue. Particles deposited in the olfactory mucosa can then undergo translocation to the olfactory bulb, where they can be directly cytotoxic or induce inflammatory and cell stress responses (Figure 1). Some combination of these mechanisms is likely to be responsible for the impaired olfactory ability among those exposed to such pollutants. Lifetime exposure to elevated levels of these pollutants may increase an individual’s risk for hyposmia or anosmia later in life. Further studies, particularly larger population surveys with individual-level pollution exposures, are needed to draw more definitive conclusions and quantify the magnitude of potential pollution effects.

## Supplemental Material

(114 KB) PDF
