# Supplementary material for: Effects of Ambient Air Pollution Exposure on Olfaction: A Review
Source: Environ Health Perspect. 2016 Jun 10;124(11):1683–93. doi: 10.1289/EHP136 (PMC5089874; doi:10.1289/EHP136)
Supplement: (114 KB) PDF [file EHP136.s001.acco.pdf]

*Environ Health Perspect*
DOI: 10.1289/EHP136

**Note to readers with disabilities:** *EHP* strives to ensure that all journal content is accessible to all readers. However, some figures and Supplemental Material published in *EHP* articles may not conform to 508 standards due to the complexity of the information being presented. If you need assistance accessing journal content, please contact ehp508@niehs.nih.gov. Our staff will work with you to assess and meet your accessibility needs within 3 working days.

# Supplemental Material

# Effects of Ambient Air Pollution Exposure on Olfaction: A Review

Gaurav S. Ajmani, Helen H. Suh, and Jayant M. Pinto

## Table of Contents

**Table S1.** Olfactory function tests
References

DOI: 10.1289/EHP136

**Table S1.** Olfactory function tests

| Test | Assessed Modality | Test Description | Reference(s) |
|---|---|---|---|
| University of Pennsylvania Smell Identification Test (UPSIT) | Odor identification | 40-item "scratch and sniff" test | Doty et al. 1984b, 1984a |
| Cross-Cultural Smell Identification Test (CC-SIT), also called the Brief Smell Identification Test (B-SIT) | Odor identification | 12-item test modified from UPSIT, specifically designed to include cross-cultural odors | Doty et al. 1996 |
| Barcelona Smell Test 24 (BAST_24) | Odor identification | 24-item test, with participants asked if they can: detect odor, recognize it, and identify it from among 4 choices | Cardesín et al. 2006 |
| San Diego Odor Identification Test (SDOIT) | Odor identification | 8-item test with participants given the same 20 choices for all 8 odors | Krantz et al. 2009; Murphy et al. 1994 |
| Sniffin' Sticks | Odor identification | 16-item test using felt-tip pens, with participants selecting correct odor from a list of 4 choices for each odor | Hummel et al. 1997 |
| | Odor threshold | Participants presented with three pens at a time, one containing n-butanol and must pick pen containing n-butanol | |
| | Odor discrimination | Participants presented three pens at a time and must pick which of three pens is different from other two | |
| 5-item Modified Sniffin' Sticks | Odor identification | 5-item test modified from Sniffin' Sticks, with participants given same 20 choices for all 8 odors | Mueller and Renner 2006 |
| Connecticut Chemosensory Clinical Research Center Test (CCCRC) | Odor identification | 10-item test with participants given the same 20 choices for all 10 odors | Cain et al. 1983 |
| | Odor threshold | Participants presented with pairs of bottles, one containing butanol and other containing blank and must determine which contains butanol | |
| Smell Diskette Test | Odor identification | 8-item test, with participant choosing correct odor from among 3 choices per odor presented as words and pictures | Briner and Simmen 1999 |

| | | | |
|---|---|---|---|
| Scandinavian Odor Identification Test (SOIT) | Odor identification | 16-item test, with participant selecting correct odor from a list of 4 choices for each odor | Nordin et al. 1998 |
| Odorant Confusion Matrix (OCM) | Odor identification | 10-item test, which participant choosing correct odor from a list of the 10 odors, with each item presented multiple times, but overall the same number of times for each item (e.g. 5 or 10) | Wright 1987 |
| T&T Olfactometry | Odor identification | 5-item test, with participants presented with progressively higher concentrations of each until able to identify odor | Fujii et al. 2002; Zusho 1983 |
| | Odor threshold | Same as odor identification test, but threshold concentration taken as level at which participant can detect odor | |
| Alinamin test | Odor identification | Thiamine propyldisulfide administered intravenously and time from administration to patient recognition of smell measured, as well as time of duration of smell | Furukawa et al. 1988 |
| Odor Stick Identification Test (OSIT) | Odor identification | 13 items specifically chosen for Japanese population, with participant choosing correct odor from 4 choices for each odor | Hashimoto et al. 2004 |

## REFERENCES


Briner HR, Simmen D. 1999. Smell diskettes as screening test of olfaction. Rhinology 37: 145–148.

Cain WS, Gent J, Catalanotto FA, Goodspeed RB. 1983. Clinical evaluation of olfaction. Am. J. Otolaryngol. 4: 252–256.

Cardesín A, Alobid I, Benítez P, Sierra E, de Haro J, Bernal-Sprekelsen M, et al. 2006. Barcelona Smell Test - 24 (BAST-24): validation and smell characteristics in the healthy Spanish population. Rhinology 44: 83–89.

Doty RL, Marcus A, Lee WW. 1996. Development of the 12-item Cross-Cultural Smell Identification Test (CC-SIT). The Laryngoscope 106: 353–356.

Doty RL, Shaman P, Dann M. 1984a. Development of the University of Pennsylvania Smell Identification Test: a standardized microencapsulated test of olfactory function. Physiol. Behav. 32: 489–502.

Doty RL, Shaman P, Kimmelman CP, Dann MS. 1984b. University of Pennsylvania Smell Identification Test: a rapid quantitative olfactory function test for the clinic. The Laryngoscope 94: 176–178.

Fujii M, Fukazawa K, Hatta C, Yasuno H, Sakagami M. 2002. Olfactory Acuity after Total Laryngectomy. Chem. Senses 27:117–121; doi:10.1093/chemse/27.2.117.

Furukawa M, Kamide M, Miwa T, Umeda R. 1988. Significance of intravenous olfaction test using thiamine propyldisulfide (Alinamin) in olfactometry. Auris. Nasus. Larynx 15: 25–31.

Hashimoto Y, Fukazawa K, Fujii M, Takayasu S, Muto T, Saito S, et al. 2004. Usefulness of the odor stick identification test for Japanese patients with olfactory dysfunction. Chem. Senses 29:565–571; doi:10.1093/chemse/bjh061.

Hummel T, Sekinger B, Wolf SR, Pauli E, Kobal G. 1997. "Sniffin" Sticks': Olfactory Performance Assessed by the Combined Testing of Odor Identification, Odor Discrimination and Olfactory Threshold. Chem. Senses 22:39–52; doi:10.1093/chemse/22.1.39.

Krantz EM, Schubert CR, Dalton DS, Zhong W, Huang GH, Klein BEK, et al. 2009. Test-retest reliability of the San Diego Odor Identification Test and comparison with the brief smell identification test. Chem. Senses 34:435–440; doi:10.1093/chemse/bjp018.

Mueller C, Renner B. 2006. A new procedure for the short screening of olfactory function using five items from the “Sniffin’ Sticks” identification test kit. Am. J. Rhinol. 20: 113–116.

Murphy C, Anderson JA, Markison S. 1994. Psychophysical Assessment of Chemosensory Disorders in Clinical Populations. In *Olfaction and Taste XI* (K. Kurihara, N. Suzuki, and H. Ogawaeds. ), pp. 609–613, Springer Japan.

Nordin S, Brämerson A, Lidén E, Bende M. 1998. The Scandinavian Odor-Identification Test: development, reliability, validity and normative data. Acta Otolaryngol. (Stockh.) 118: 226–234.

Wright HN. 1987. Characterization of olfactory dysfunction. Arch. Otolaryngol. Head Neck Surg. 113: 163–168.

Zusho H. 1983. Olfactometry in Japan. Rhinology 21: 281–285.
